# Pollution pressure drives microbial assemblages that improve the phytoremediation potential of heavy metals by *Ricinus communis*

**DOI:** 10.1007/s11274-024-04025-8

**Published:** 2024-06-13

**Authors:** Daniela Rubio-Noguez, Luz Breton-Deval, Ilse Salinas-Peralta, Katy Juárez, Leopoldo Galicia

**Affiliations:** 1 Instituto de Biotecnología, Universidad Nacional Autónoma de México, Avenida Universidad 2001, Colonia Chamilpa, Cuernavaca, Morelos 62210 México; 2 Consejo Nacional de Ciencia y Tecnología, Avenida de los Insurgentes Sur 1582, Crédito Constructor, Benito Juárez, Ciudad de México, 03940 México; 3 Instituto de Geografía Investigaciones en Ecosistemas y Sustentabilidad, Universidad Nacional Autónoma de México, Investigación Científica, Ciudad Universitaria, C.U., Ciudad de México, CDMX, 04510 México

**Keywords:** Phytormediation, Heavy metals, Contaminated soils, Microbial communities, Environmental adaptation

## Abstract

Due to the rapid expansion of industrial activity, soil pollution has intensified. Plants growing in these polluted areas have developed a rhizobiome uniquely and specially adapted to thrive in such environments. However, it remains uncertain whether pollution acts as a sufficiently selective force to shape the rhizobiome, and whether these adaptations endure over time, potentially aiding in long-term phytoremediation. Therefore, in the present study, we aimed to compare whether the microbiome associated with roots from plants germinated in polluted riverbanks will improve the phytoremediation of Cd and Pb under mesocosm experiments compared with plants germinating in a greenhouse. The experimental design was a factorial 2 × 2, i.e., the origin of the plant and the presence or absence of 100 mg/L of Cd and 1000 mg/L of Pb. Our results showed that plants germinated in polluted riverbanks have the capacity to accumulate twice the amount of Pb and Cd during mesocosm experiments. The metagenomic analysis showed that plants from the river exposed to heavy metals at the end of mesocosm experiments were rich in *Rhizobium sp. AC44/96 and Enterobacter sp.* EA-1, *Enterobacter soli*, *Pantoea rwandensis, Pantoea endophytica.* In addition, those plants were uniquely associated with *Rhizobium grahamii*, which likely contributed to the differences in the levels of phytoremediation achieved. Furthermore, the functional analysis revealed an augmented functional potential related to hormones, metallothioneins, dismutases, and reductases; meanwhile, the plants germinated in the greenhouse showed an unspecific strategy to exceed heavy metal stress. In conclusion, pollution pressure drives stable microbial assemblages, which could be used in future phytostabilization and phytoremediation experiments.

**Graphical Abstract:**

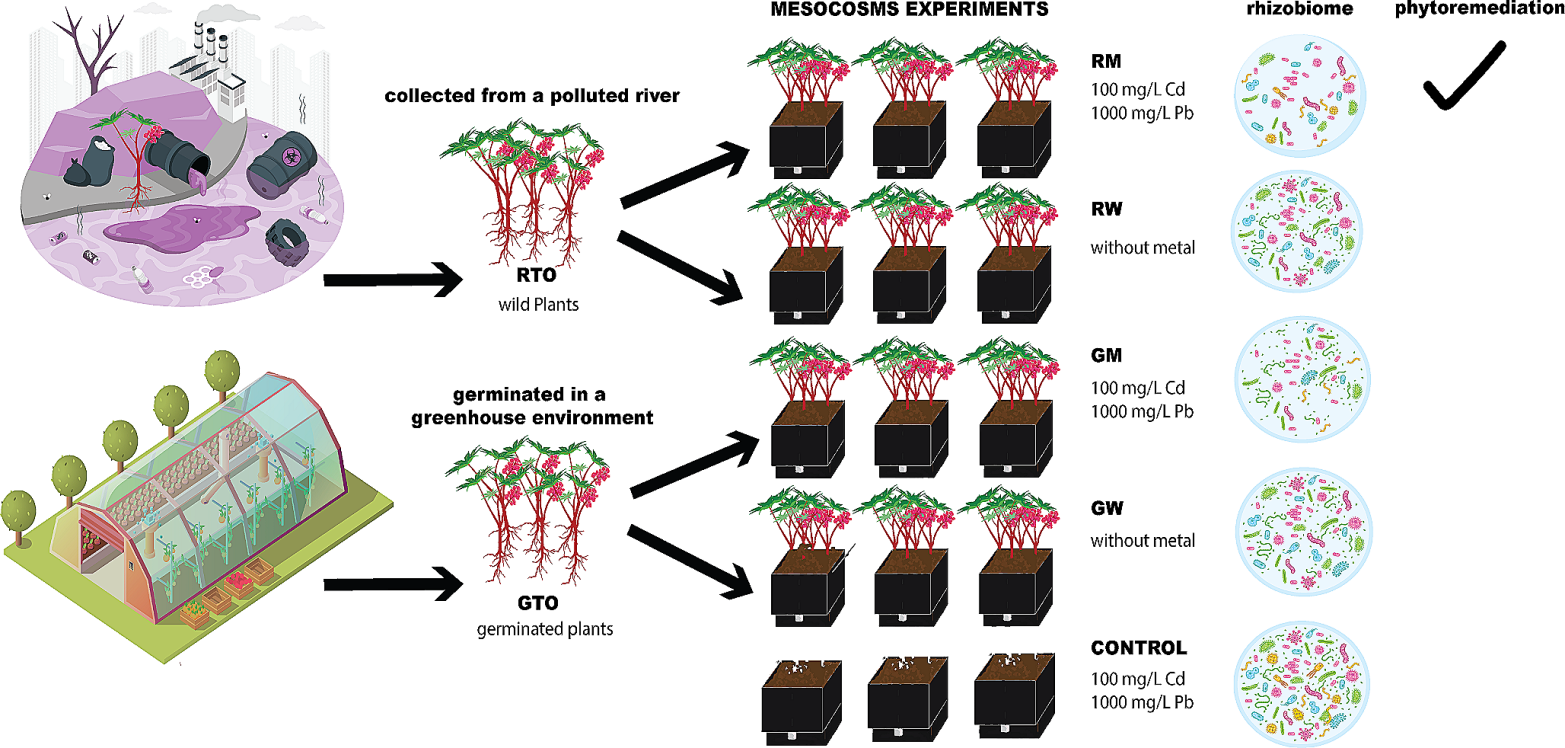

## Introduction

Over the past few decades, heavy metals have reached alarming environmental levels due to human activities, such as mining and industrial processes. Improper handling and waste disposal of these metals have resulted in pollution, habitat loss, reduced biodiversity, climate change, and health problems (Aini et al. 2023). The topic of heavy metal pollution has garnered substantial environmental concern due to the persistence of these elements which have been accumulating in soils since 19th century, with the potential to bioaccumulate and pass through the food chain (Ozaki et al. 2019; Gabrielli et al. 2020; Andleeb et al. 2023). In this regard, phytoremediation is a highly effective approach for removing heavy metals. This cost-effective technique uses plants and their associated microorganisms to extract contaminants and/or detoxify contaminated systems by inactivating or translocating toxic substances (Bhanse et al. 2022). The effectiveness of phytoremediation relies on several factors, including the degree of contamination, the type of contaminant, the bioavailability of the pollutant, the type of plant used, etc., but above all, the microbial communities associated with the rhizosphere (rhizobiome) have a vital role in the success of the phytoremediation (Chen et al. 2018b; Mudgal et al. 2023). Phytoremediation studies experienced a significant surge in interest during the 1990s. Research in this field continues to advance, leveraging novel technologies such as metagenomics. These cutting-edge tools enable researchers to uncover new insights and enhance the effectiveness of phytoremediation (Cunningham et al. 1995; Zhang et al. 2024).

It is well known that environmental conditions shape the microbial community; for example, pollution is a stress factor that enriches the microbial community in genera with the metabolic capacity to be successful in that environment (Almasia et al. 2016; Qin et al. 2022; Zhao et al. 2023). Accordingly, several studies have proposed that the rhizobiome of plants from polluted sites with heavy metals are rich in *Pseudomonadota*, *Actinomycetota*, and *Chloroflexota*. Some members of those phyla enhance the bioavailability of heavy metals by producing secondary metabolites, such as siderophores, which modify the medium by promoting the chelation and solubilization of heavy metals and influencing their translocation in the plant or increasing the production of biosurfactants (Liu et al. 2022; de Lima et al. 2022; Yadav et al. 2023). Advancements in phytoremediation research have focused on isolating key microbial consortia with metabolic capacities to remove pollutants (Chen et al. 2018b). However, several factors need to be addressed for the success of bioaugmentation using this approach. These include ensuring that the bioaugmented microorganisms exhibit rapid growth, resist high concentrations of contaminants, and can compete effectively against indigenous bacteria, among other considerations (Montreemuk et al. 2024).

To boost phytoremediation rates of pollutants, a promising strategy could entail harnessing the entire microbial community previously acclimated to the specific pollutant. Furthermore, (Jiang et al. 2022) and (Jousset and Lee 2023) propose the term rhizosphere microbiome transplant (RMT), homologating the technique used in health research called fecal microbiome transplantation, where the gut microbiome from healthy donors to patients is used to improve the health of patients; however, studies about the RMT are scarce. For example, (Bziuk et al. 2022) utilized RTM to improve the resistance rate of *Hordeum vulgare* to the fungus *Blumeria graminis* infection, and (Jiang et al. 2022) to enhance the resistance of Solanaceae against *Ralstonia solanacearum*. RMT has been tested to improve the resistance against diseases. Therefore, the question remains whether polluted environments are a selective pressure intense enough to modify the rhizobiome and impact the phytoremediation potential and whether this microbial core is stable even if the plant is moved from that site. Because if we can preserve the bacterial core and metabolic potential of plants, we can use that potential to inoculate more plants using that core.

In order to answer those questions, we selected the Apatlaco riverbanks as a pollutant environment. This basin has been the subject of an ongoing investigation due to pollution resulting from the receipt of various discharges. As a consequence, the river has experienced elevated levels of a wide range of pollutants, including chemical oxygen demand (COD), ammonia, cadmium (Cd), lead (Pb), and the presence of bacterial genera such as *Acinetobacter*, *Myroides, Aeromonas*, among others (Breton-Deval et al. 2019). Therefore, in the present study, we aimed to test the following hypothesis: The rhizobiome from plants germinated in the Apatlaco riverbanks will have the metabolic potential to improve the phytoremediation of Cd and Pb compared with plants germinated in a greenhouse. The objectives are: (i) To assess the composition and diversity of the rhizobiome associated with plants germinating in the Apatlaco riverbanks and plants in a greenhouse. (ii) To compare the composition and diversity of the rhizobiome in different conditions. (iii) To compare the efficiency of phytoremediation of Cd and Pb between plants from the Apatlaco riverbanks and plants from the greenhouse environment. (iv) To evaluate the functional potential underlying the differences in phytoremediation efficiency between the conditions through metagenomic analyses.

We selected *R. communis*, a shrubby plant that grows 1 to 5 m tall and develops quickly in diverse habitats, to carry out these experiments. This plant has been extensively researched for its ability to accumulate high concentrations of a wide range of heavy metals, such as Cd > Cu > Zn > Pb, with promising results, and it is widespread in the riverside of polluted Apatlaco River (Bauddh et al. 2015).

## Materials and methods

### Sampling and collection of wild plants

We collected 32 individuals of *R. communis* (30 ± 7 cm tall) growing on the bank of Apatlaco River (18˚51ʹ6ʺ N, 99˚13ʹ58ʺ W) to implement the mesocosms. The plants underwent a 2-week acclimatization period before starting the phytoremediation experiments. A set of 4 plants from this collection were digested to analyze their heavy metal. These plants from the river were called RT0.

### The seeds plant germination process in greenhouse conditions

First, 40 previously collected *R. communis* seeds were thoroughly washed and disinfected using a solution of NaCl (10%) and TritonX100 (0.02%). The washing process was repeated three times to ensure the seeds were impurities-free. Next, a chemical scarification process was conducted by immersing the seeds in H_2_SO_4_ for 10 min to remove the seed coat. After the allotted time, the scarified seeds were allowed to rest in sterile distilled water for approximately 24 h to facilitate the imbibition process before planting. The germination soil consisted of a sieved mixture of prepared and black soil in a ratio of 70:30 w/w. This soil mixture was sterilized in an autoclave for 15 min using a 5 L aluminum container. The seeds were then sown at a depth of approximately 1.5 cm. After about two weeks, a 1 cm thick layer of worm humus was added to provide essential nutrients for plant growth. Once the seedlings had developed their first true leaves, they were carefully transplanted into compostable pots to support further growth and facilitate their eventual transfer into the Dutch pots for the experiment. When the plants reached a height of 20 cm, they were ready to commence the experiments. These plants were called GT0.

### Phytoremediation potential experimental design and mesocosms implementation

**Fig. 1:**
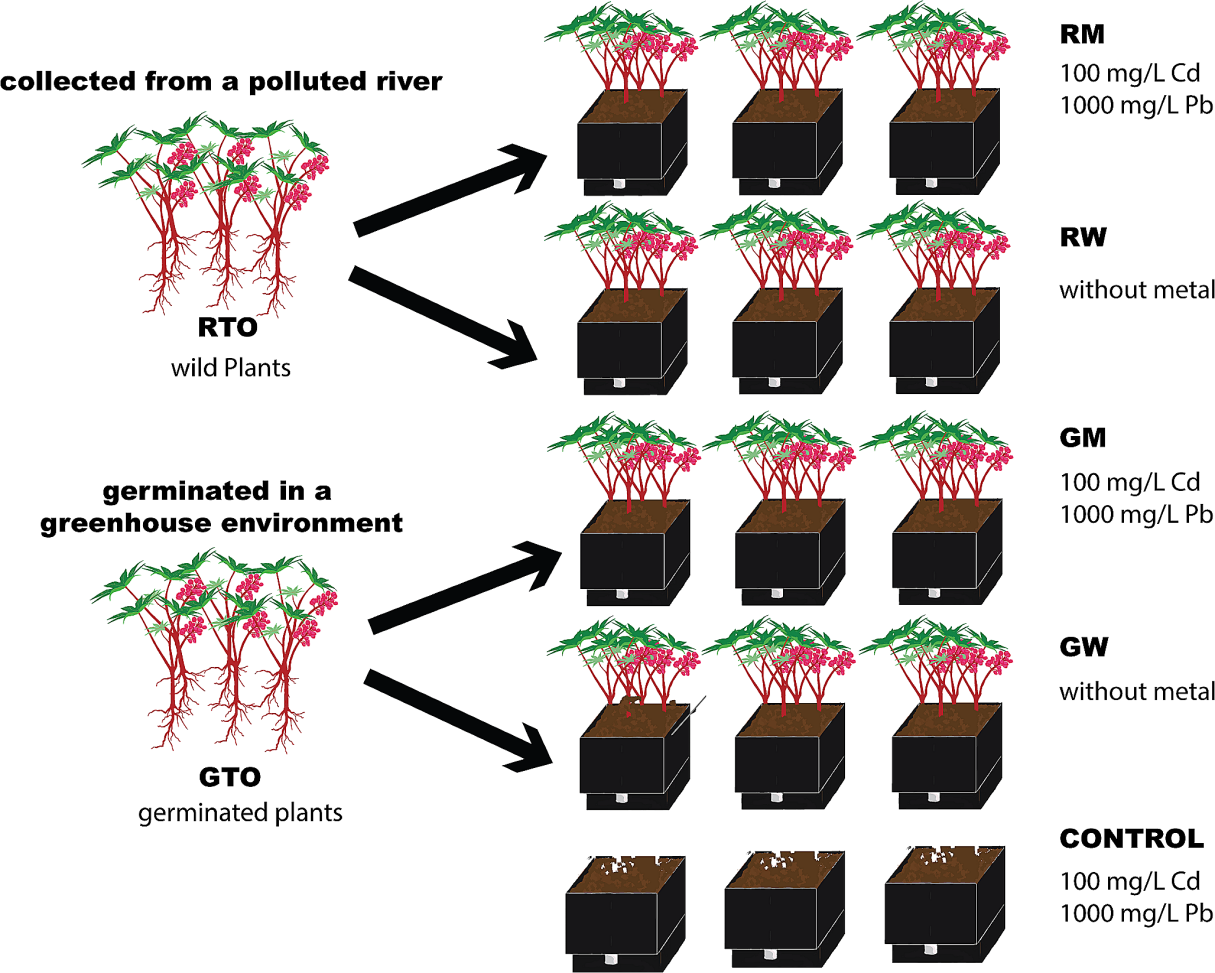
The experimental design was a factorial 2 × 2, where factors were the origin of the plant and the presence or absence of the following heavy metals: Cd and Pb. To carry out the experiment we implemented mesocosms comprised of seven blocks: three blocks consisting of *R. communis* germinated in greenhouse environment (GT0) watered with heavy metal (GM) and without heavy metals (GW), three blocks consisting of *R. communis* collected from the river (RT0), with heavy metals (RM) and without (RW) and one block served as the soil control

The experimental design was a factorial 2 × 2, where factors were (1) the origin of the plant (RT0 or GT0) and (2) the presence or absence of heavy metals (Cd and Pb), see Fig. 1. To carry out the phytoremediation experiment, we implemented mesocosms comprised of four conditions with triplicate; every replicate has 4 plants as a result, we evaluated 12 plants per condition. The conditions were: (i) *R. communis* germinated in a greenhouse environment watered three times per week with heavy metal mix (GM, from the greenhouse with metal) and (ii) *R. communis* germinated in a greenhouse environment watered three times per week with water (GW, from greenhouse without metals), (iii) *R. communis* collected from the river, watered three times per week with heavy metal mix (RM, from the river with metal) and IV) *R. communis* collected from the river, watered three times per week with water (RW, from the river without metal) and V) soil without plant watered three times per week with heavy metal mix to know the heavy metal removal associated with physical conditions which ranged around 1%. Depending on the treatment requirements, the mesocosms were watered with tap water or a solution containing cadmium nitrate tetrahydrate and lead (II) nitrate (Sigma-Aldrich) with a heavy metal concentration of 100 and 1000 mg/L, respectively.

Each condition consisted of three Dutch pots with a capacity of 33 L, each containing four plants. The potting mixture comprised equal parts of tezontle, gravel, and soil. The soil conditions included a pH of 5.7, an organic carbon content of 9.14%, and a cation exchange capacity of 53.94 cmolc/kg. Additionally, the soil moisture was maintained at 60%. Throughout the experiment, plants were kept in a greenhouse with a temperature of 26 ± 5.98 °C and a relative humidity of 64.42 ± 3.93%.

### Phytoremediation efficiency

The heavy metals concentrations were analyzed in samples at the start and end of the experiment utilizing inductively coupled plasma optical emission spectrometry (Variant 720-ES ICP-OES System). First, the plant samples were digested in the laboratory, adding 4 ml of HNO_3_ and 2 ml of H_2_O_2_ (30% v/v in water), followed by a 60-minute heating process. Afterward, the samples were filtered through the Whatman 42 filter paper to remove solid particles or impurities. All samples were analyzed by duplicate, and the results were used to calculate: 1. Bioaccumulation Factor (BF). BF is defined as the number of heavy metals accumulated in the plant’s root and aerial parts relative to the concentration of the metal present in the soil (Formula 1) (Arthur et al. 2022). The Translocation Factor (TF) describes the relationship between the amount of HM moved from the roots to the shoots (Formula 2).

1 $$BF = \frac{HM_{plant}}{HM_{soil}}$$

2 $$TF = \frac{HM_{shoots}}{HM_{roots}}$$

### DNA extraction and sequencing

Before starting the phytoremediation experiments, we analyzed the rhizosphere microbiome (rhizobiome) present in groups GT0 and RT0 to understand every group’s biodiversity and functional capabilities. The rhizospheric DNA extraction followed the (Bulgarelli et al. 2012) method with particular modifications to start, we created a composite sample by pooling all the plants from each experimental condition to create a single metagenomic sequence representative of each condition. Roots were gently agitated in 5 ml of PBS buffer for approximately 5 min until a substantial amount of adhering soil settled. After this, the samples underwent centrifugation at 10,000 rpm for 1 min to form a pellet of rhizospheric soil. We took 100 mg of this pellet plus 150 µl of PBS buffer for DNA extraction using the DNAeasy PowerWater kit (QIAGEN), by the manufacturer’s instructions. The integrity of each extraction was assessed through a Qubit 2.0 ss-DNA kit. Subsequently, an Illumina library was generated for each sample using the TruSeq kit V2 (Illumina, Inc., San Diego, CA, USA), strictly adhering to the manufacturer’s specifications. This library preparation maintained an average fragment size of 500 bp. The sequencing was done on the NovaSeq 6000 platform (Illumina, Inc., San Diego, CA, USA) by Novogene Corporation Inc, CA.

### Bioinformatic and statistical analysis

The quality control analysis, adapter trimming, and elimination of low-quality sequences were carried out using the FastP program (Chen et al. 2018a). The tool to profile the microbial communities was executed using metagenomics phylogenetic analysis (MethaPhlAn4) (Blanco-Míguez et al. 2023). We assembled the raw reads using Megahit, while gene prediction and annotation were accomplished through Prodigal (Hyatt et al. 2010) and DIAMOND (Buchfink et al. 2021). The Kyoto Encyclopedia of Genes and Genomes (KEGG) was used to search for the proteins identified in the samples (Kanehisa et al. 2017). The orthology numbers (KO) were configured on the KEEG Mapper website to identify the related pathways. The raw reads derived from the whole metagenome sequencing were deposited at the National Center for Biotechnology (NCBI) information database under the BioProject number PRJNA1017462.

All statistical analysis was carried out using R and Rstudio. It started with a Welch’s *t*-test analysis of the heavy metals accumulated in the different plant parts between the treatments. This was followed by the alpha diversity and the NMDS analysis, which explains the complex interaction between selective pressures caused by metals and their subsequent impact on community similarities and differences. The arrangement of samples on the NMDS plot offers a valuable understanding of how the microbiome reacts to these environmental influences. Furthermore, we include a Linear Discriminant Analysis (LDA), or LEfSe, to identify the characteristics most likely to account for class distinctions. This technique combines a conventional statistical significance test with an extra assessment that incorporates biological coherence and the significance of the effect (Segata et al. 2011).

## Results and discussion

### Rhizobiome richness and composition of plants RT0 and GT0

The rhizobiome of GT0 was more diverse than RT0, as shown by Shannon/Simpson index of 4.06/0.9738 versus 2.78/0.8267, respectively. The rhizobiome of RT0 plants was particularly enriched in *Acinetobacter johnsonii* (54%), which is attributed to the pollution in the watershed where the plants were collected (Breton-Deval et al. 2019). This potential opportunistic pathogen could be introduced into the system through one of the multiple municipal discharges and found to thrive in optimal conditions. These results are consistent with (Jia et al. 2021), where *Acinetobacter johnsonii* was isolated from a polluted river line and found a correlation between its resistance phenotype and the presence of heavy metals such as Pb and Cd in the site. Furthermore, the sample RT0 was rich in diverse species of *Acinetobacter*, such as *Acinetobacter cumulans* (6%), isolated and described from hospital sewage with the ability to acquire and cumulate diverse resistance determinants (Qin et al. 2019). *Acinetobacter gandensis* (3%) was isolated from horse dung (Smet et al. 2014), and *Acinetobacter towneri* (4%), a well-known bacterium from water environments and a recognized reservoir of antimicrobial resistance genes (Maehana et al. 2021). Many other species exhibit remarkable abilities to synthesize various polymeric substances, such as *Cloacibacterium normanense* (3%), which can synthesize extracellular polymeric substances capable of removing high concentrations of metals (Nouha et al. 2016) As with *Sphingobacterium mizutaii* (3%) (Burgos-Díaz et al. 2011), *Pseudoxanthomonas mexicana* (2%) (Nayak et al. 2009), and *Chryseobacterium sp.* VAUSW3 (3%) (Hu et al. 2022). Other examples of bacteria found in those plants are *Novosphingobium tardaugens* (1%), an aerobic bacteria known for its ability to degrade polycyclic aromatic hydrocarbons and remove heavy metals from polluted sites, making it a promising candidate for bioremediation projects (Fujii et al. 2003).

**Fig. 2:**
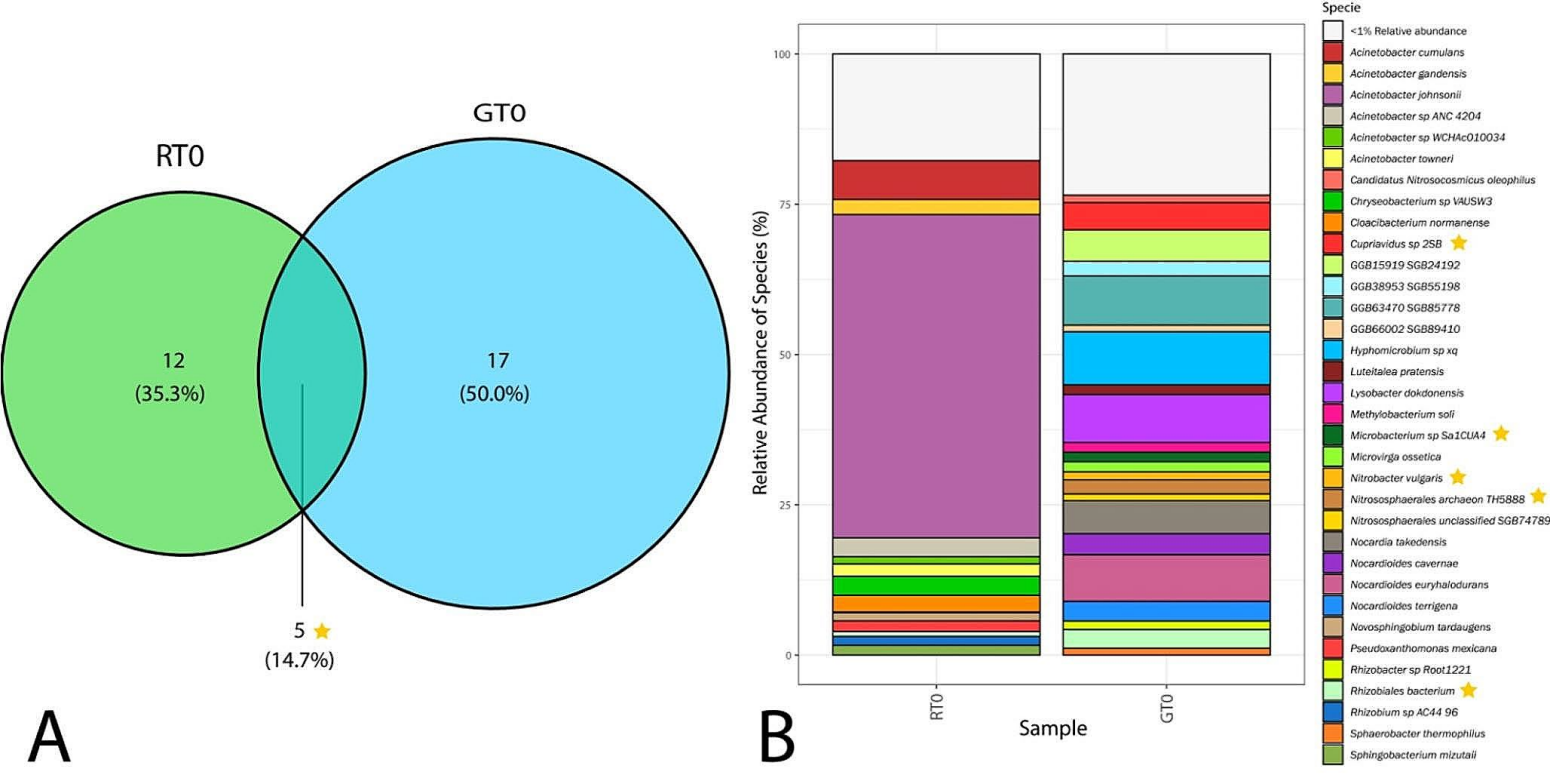
**A** Venn diagram illustrates how many species with a relative abundance ˃ 1% of relative abundance share RT0 with GT0, they only share 5 species. **B** Composition and diversity of the rhizobiome associated with RT0 and GT0.

In contrast, the rhizobiome in GT0 was rich in microorganisms involved in significant processes commonly found in soil see Fig. 2. For example, certain species of bacteria promote plant growth, such as *Methylobacterium soli* (2%) and *Microvirga ossetica* (2%) (Chauhan et al. 2015; Msaddak et al. 2019). Furthermore, other bacteria play a role in the nitrogen cycle, such as *Nitrososphaerales archaeon* (2%) and *Nitrosocosmicus oleophilus* (1%), both of which contribute towards ammonia oxidation, as well as *Nitrobacter vulgaris*, which is a nitrite-oxidizing bacteria (1%) and *Hyphomicrobium sp* (9%) a denitrification bacterium that participate in the conversion of nitrates into nitrogen gas (Urakami).

Some other examples include *Lysobacter dokdonensis* (8%), *Luteitalea pratensis* (2%), and *Cupriavidus sp.* (5%), which produces several types of antimicrobial chemicals that protect the plant against pathogens (Ren et al. 2023). GT0 plants were enriched with various species of the *Nocardioides* genus, which belong to the Actinobacteria class. This type of bacteria is typically found in different kinds of soils; for example, *Nocardioides euryhalodurans* (8%) was isolated from sandy soil in Korea (Roh et al. 2020), while *Nocardioides cavernae* (3%) (Han et al. 2017) was discovered in a karst cave in China. Additionally, *Nocardioides terrigena* (3%) (Yoon et al. 2007) and *Nocardia takedensis* (12%) (Lotte et al. 2020) have recently been identified as a pathogen.

### Phytoremediation of Pb and Cd using *R. communis*

**Fig. 3:**
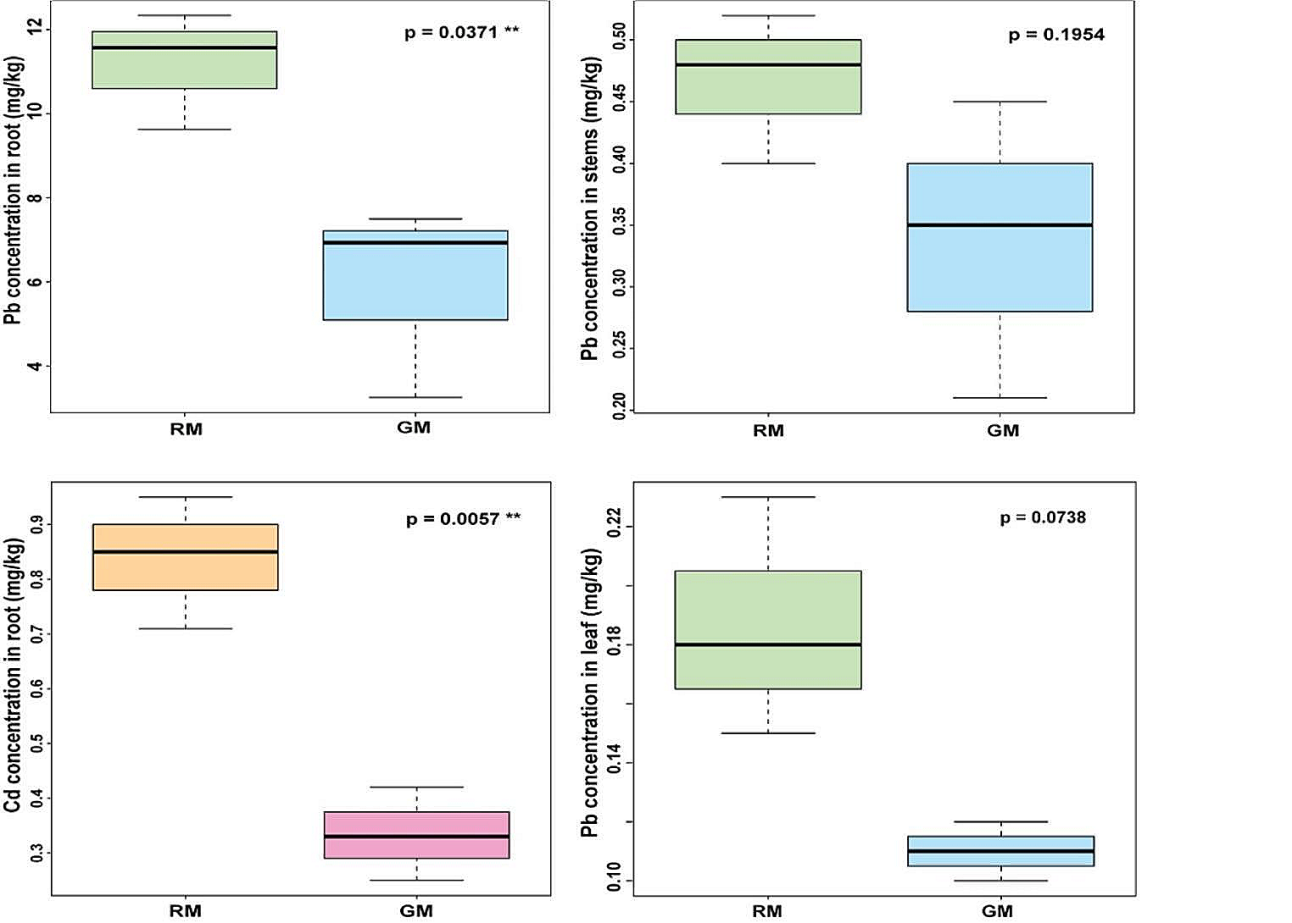
Phytoremediation of lead (Pb) and Cadmium (Cd) using Ricinus communis: Utilizing Green/Blue combination for Pb removal and investigating Cd removal with the Pink/Orange combination, with a focus on Cd concentration in roots due to significance. The Pb concentrations in stems and leaves did not reach statistical significance (*p* < 0.05), and no accumulated concentration of Cd was detected on the leaves

The RM plants exhibited significantly higher levels of Pb accumulation in roots (11.18 ± 3.91 mg/kg) compared to the accumulation in GM roots (5.89 ± 3.31 mg/kg). The accumulation of Pb in stems and leaves did not show statistical significance between both treatments (p ˂ 0.05) Fig. 3. RM plants accumulated significantly more Cd in roots (*p* < 0.05) with levels of 0.84 ± 0.17 mg/kg compared to GM roots (0.33 ± 0.04 mg/kg (Fig. 3). The accumulation of Cd in stems was 0.08 ± 0.04 mg/kg and 0.06 ± 0.01 mg/kg for RM and GM but the difference between both treatments did not reach statistical significance (*p* < 0.05), respectively, no accumulation was detected Cd in leaves. The Bioaccumulation Factor (BF) is defined as the correlation between the quantity of HMs accumulated in various plant components and the concentration of HMs in the soil (Sanjosé et al. 2022), RM showed a BF of 1.79 for Pb. At the same time, GM exhibited a BF of 0.94. For Cd, the BF values were 1.23 for RM and 1.01 for GM.

Considering the translocation of heavy metals, a crucial aspect of phytoremediation, this mechanism involves phytovolatilization and phytoextraction as plants transfer metal ions from roots to shoots (Jhanani et al. 2023). There is a significant disparity in the amount of Pb and Cd accumulated in wild plants compared to germinated plants. The Pb translocation factor (TF) between RM and GM were 0.04 and 0.10, respectively. The TF for Cd followed a similar trend, with values of 0.10 for RM and 0.18 for GM. Plants with a BF value greater than 1 and a low TF value are known to be suitable for phytostabilization (Cheraghi et al. 2011). These results demonstrated a significant disparity in the amount of Pb and Cd accumulated in RM compared to GM. This increase in HM accumulation in RM plants may be attributed to the coupled rhizobiome, which potentially enhances the solubility and bioavailability of HMs in the soil. Therefore, *R. communis*, could be used for phytostabilization of both pollutants.

### The rhizobiome of *R. communis* at the end of phytoremediation mesocosm

**Fig. 4:**
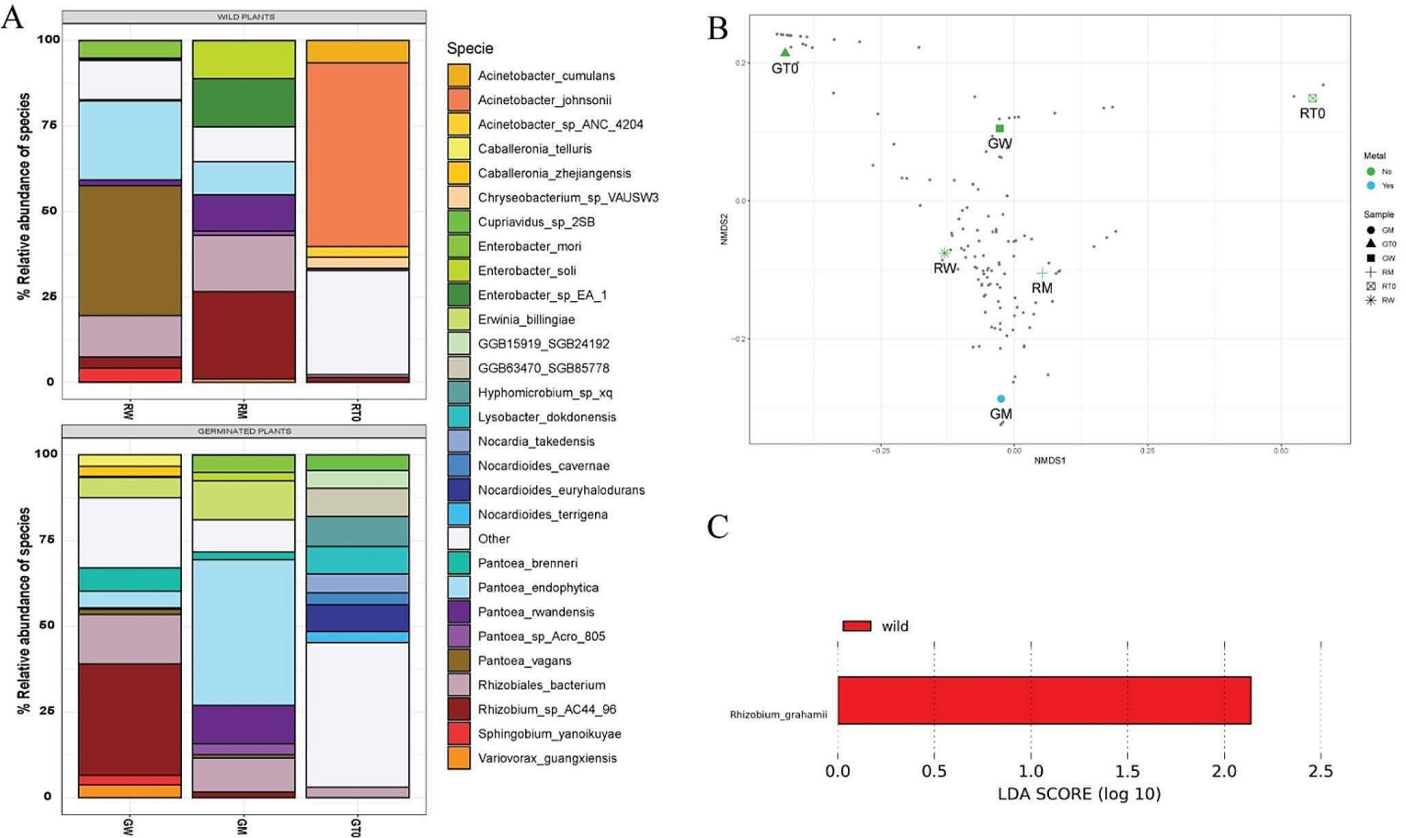
The microbial community after the phytoremediation eAperiments. **A** Taxonomic composition of root-associated bacterial communities (rhizobiome) from the different treatments after the phytoremediation experiment. **B** NMDS analysis with all the treatments. 4 C LEfSe analysis to identify relevant microorganisms at the end of the phytoremediation

Regarding rhizobiome analysis, the Shannon/Simpson alpha diversity values for RW were calculated at 3.63/0.88, while those for the RM yielded values of 2.84/0.92. Notably, it becomes evident that the metal-free RW sample demonstrates a more pronounced species diversity when compared to RM, exhibiting a more balanced distribution of species abundances, as corroborated by the Simpson Index. Meanwhile, for germinated plants, the Shannon/Simpson indices were determined to be 3.21/0.92 for GW and 2.71/0.88 for GM. These observations align with the trend observed in wild plants, whereby the presence of heavy metals corresponds to diminished species diversity when contrasted with non-exposed plants (Fig. 4A). Plants without heavy metals exposure, RW and GW, showed differences between their alpha diversity values 3.63/0.88 and 3.21/0.92 respectively, where RW exhibited slightly higher values, however, in order to evaluate if those changes represent a real difference, more data needs to be assessed, perhaps the difference is not significant, and the values showed a microbial community without stress.

Upon examining the NMDS plot (Fig. 4B), a distinct pattern becomes evident, highlighting a more pronounced similarity between the communities of GW and RW samples compared to those of RM and GM samples. This pattern underscores the influence of metal selective pressures in shaping the composition of these communities. Notably, the RM and GM samples are closer to each other within the NMDS plot, further substantiating the notion of metals exerting a discernible impact on community dynamics.

When comparing the lab-germinated samples (GM and GW), a more pronounced spatial disparity becomes evident in contrast to those originating from the river environment (RM and RW). This phenomenon might be attributed to the fact that the river-associated microbiome has already undergone exposure to the selective pressures of heavy metal pollution, potentially resulting in a more stabilized community structure. Consequently, the variations in community composition are comparatively less pronounced among river-originating samples.

The RT0 microbiome, as previously elucidated, exhibited a pronounced prevalence of *Acinetobacter*. However, noteworthy findings surfaced upon the conclusion of the experiments. Specifically, significant enrichment of specific microbial species became evident in different contexts. RM exhibited a marked enrichment of *Rhizobium sp AC44/96* (26%), a bacterium recognized for its capacity to establish mutualistic symbiosis with plant roots (Fig. 4A). This symbiotic relationship is pivotal in agriculture due to its nitrogen-fixing ability, which enhances essential nutrient availability for plants. Additionally, the enriched presence of several members from the Enterobacteriaceae family was observed in these plants, including *Enterobacter sp. EA_1* (14%) and *Enterobacter soli* (11%), the latter being recently acknowledged for its ability to thrive in polluted soil environments (Manter et al. 2011). An enrichment of *Pantoea* genus members, such as *Pantoea rwandensis* (11%) and *Pantoea endophytica* (10%), was also observed. *Pantoea*, a diverse bacterial genus, thrives across habitats and ecological niches. Certain strains within this genus are recognized as plant growth-promoting bacteria (PGPB) due to their nitrogen synthesis, ammonia and phosphorus solubilization, and iron sequestration through bacterial siderophores (Lorenzi et al. 2022). Moreover, *Pantoea* displays inhibitory properties against plant pathogens by producing antibiotic enzymes and inducing systemic resistance (Lekired et al. 2023). Other species identified in the rhizobiome of RM included some PGPB bacteria like *Paraburkholderia tropica* (3%) (Ramirez-Villacis et al. 2023), *Variovorax guangxiensis* (1%) (Gao et al. 2015), and *Streptomyces mirabilis* (1%) (Okazaki et al. 2021). In contrast, GM displayed a substantial portion (approximately 58%) of their microbial species belonging to the *Pantoea* genus. Notably, *Pantoea endophytica* (43%), *Pantoea rwandensis* (11%), *Pantoea brenneri* (2%), *Pantoea conspicua* (1%), and *Pantoea vagans* (1%) demonstrated beneficial characteristics as described earlier. *Erwinia bilingiae* (11%), recognized for its exopolysaccharide production and iron uptake mechanisms (Kube et al. 2010), and *Rahnella variigena* (3%), *Enterobacter mori* (5%) and *Enterobacter soli* (2%) a plant-growth-promoting bacteria (Mitra et al. 2020), were also identified.

Furthermore, RW exhibited a microbial community rich in species such as *Pantoea vagans* (38%), *Pantoea endophytica* (23%), *Enterobacter mori* (5%), *Sphingobium yanoikuyae* (4%), *Novosphingobium panipatense* (2%) *Pantoea rwandensis* (2%), *Mycobacterium sp* (1%) and *Shinella kummerowiae* (1%). The RW profile closely resembles GM’s, exhibiting a substantial presence of the *Pantoea* genus. Nonetheless, the profile retains some species, possibly originating from the river’s assembly, including well-established bacteria like *Sphingobium yanoikuyae*, known for its proficiency in breaking down various saturated hydrocarbons and aromatic compounds (Gupta et al. 2009), as well as *Novosphingobium panipatense*, recognized for its effectiveness in degrading phenanthrene, anthracene, and naphthalene.

Meanwhile, GW presents a rhizobiome rich in *Rhizobium_sp_AC44_96* (33%). This bacterium enhances essential nutrient availability for plants and other species previously described, such as *Pantoea brenneri* (7%), *Erwinia billingiae* (6%), *Pantoea endophytica* (5%), *Variovorax guangxiensis* (4%), *Caballeronia telluris* (3%), *Caballeronia zhejiangensis* (3%), *Sphingobium yanoikuyae* (3%), *Stenotrophomonas spp.* (3%), *Pseudomonas fluorescens* (2%), and *Pantoea vagans* (1%). A difference between this profile and the others is the number of species and the appearance of some new species like *Caballeronia telluris*, *Stenotrophomonas* spp., and *Pseudomonas fluorescens.*

It is evident that most samples share similar microorganisms, with disparities primarily arising from differences in their proportional representation. Leveraging the LEfSe, a rigorous statistical method accounting for microorganism proportions and relevant environmental variables, identified *Rhizobium grahamii* as uniquely significant to RM sample (Fig. 4C). This species demonstrated increased abundance in the metal-exposed sample (RM). *Rhizobium grahamii* possesses the largest reported chromosome among *Rhizobium* species, with approximately 5,400 kbp, including a segment of about 1,073 kbp linked to a genomic island potentially originating from plasmid integration or an Integrative and Conjugative Element (ICE). This segment encodes proteins associated with chemotaxis, DNA metabolism, ABC transporters, and other functions, potentially contributing to its success in such an environment (Althabegoiti et al. 2014).

### The functional potential of the rhizobiome in cd and pb phytoremediation

**Fig. 5:**
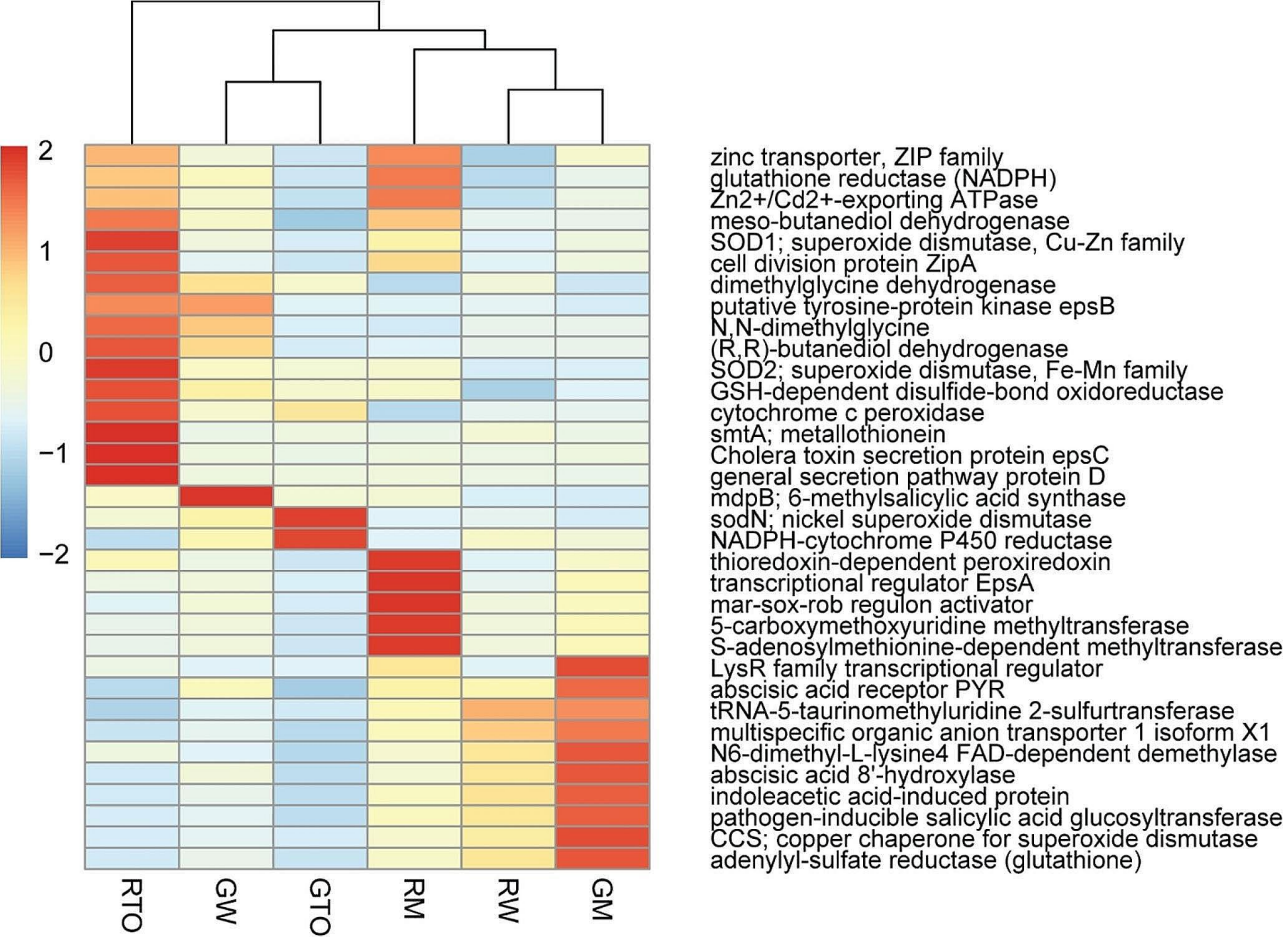
The potential metabolic pathways exhibited by the rhizobiome under each condition during the phytoremediation experiments

The analysis of the functional gene profile has revealed distinct molecular and cellular function patterns within the rhizobiome following the completion of the heavy metal exposure period. Figure 5 illustrates the potential metabolic pathways exhibited by the rhizobiome under each condition during the phytoremediation experiments. While metatranscriptomic analyses could confirm this assertion, assessing functional potential enables us to identify pathways of interest that could be further elucidated using various molecular tools. The primary mechanisms microorganisms employ to mitigate the toxicity of cationic metals, such as Cd and Pb, involve metal efflux, sequestration, DNA repair systems, and adjustments in membrane fluidity (Breton-Deval et al. 2022). However, as observed, root-associated microorganisms exhibit varying functional potentials, some more specific than others. Microorganisms associated with plant roots from the river referred to as RT0, show a range of elements. Terpene syntheses are a critical enzyme in producing volatile organic compounds (VOCs). VOCs have the potential to stimulate microbial activity, attracting new beneficial microorganisms that support the process or providing a substrate for the increase of bacterial biomass (Liu et al. 2023). Furthermore, VOCs can reduce plant susceptibility to some diseases (Gan et al. 2023).

Additionally, RT0 rhizobiome presented certain reductase enzymes, such as cytochrome c peroxidase peroxidases, exopolysaccharides, and metallothioneins. The RM rhizobiome profile focused more on metallothioneins, exopolysaccharides, and dismutases. On the other hand, the RW rhizobiome profile exhibited a similarity to the GM profile, almost as if the initial exposure to stressors like heavy metals prompted a broader and more generalized response. On the contrary, microorganisms linked to GT0 plants exhibit a functional capacity predominantly focused on dismutases and peroxidases. As shown in Fig. 5, there is a noticeable clustering between GT0 and GW, implying analogous profiles as these plants have not been subjected to heavy metal stress. In contrast, microorganisms associated with GM, originating from GT0, display a discernible pattern distinct from their source. This pattern reveals an augmented functional potential related to hormones, metallothioneins, dismutases, and reductases.

## Conclusion

Pollution could be a force that decreases biodiversity, enriching some species with the metabolic capacities to allow them to cope with a contaminated environment, as we can observe after analyzing the rhizobiome germinated in the polluted riverbank and the greenhouse. After the phytoremediation analysis, plants germinated in the riverbank (RM) exhibited a significantly higher Pb and Cd accumulation than greenhouse-germinated plants (GM). The accumulation trend was consistent across roots. The BF values for RM indicated a significant potential for heavy metal uptake compared to GM. The TF values demonstrated RMs suitability to phytostabilization, suggesting its applicability in phytoremediation efforts. Our experiment underscored that polluted environments constitute a potent, selective pressure capable of modifying the rhizobiome and influencing phytoremediation potential.

Furthermore, it revealed the stability of the microbial core even post-relocating the plant. Preserving this bacterial core and its metabolic potential could serve as a valuable strategy for inoculating more plants. The symbiotic rhizobiome of RM plants likely influenced the solubility and bioavailability of heavy metals, thereby facilitating their increased accumulation in plant tissues. Notably, *Rhizobium grahamii*, enriched in RM, may play a significant role in metal accumulation, given its genomic attributes associated with metal tolerance and DNA repair mechanisms.

## Data Availability

No datasets were generated or analysed during the current study.
